# The role of alexithymia and autistic traits in predicting quality of life in an online sample

**DOI:** 10.1016/j.rasd.2021.101887

**Published:** 2022-02

**Authors:** David Mason, Francesca Happé

**Affiliations:** Social, Genetic and Developmental Psychiatry Centre, King’s College London, UK

**Keywords:** Autism spectrum disorder, Autistic traits, Quality of life, Alexithymia, Depression, Anxiety

## Abstract

**Background:**

Autistic people tend to report poorer Quality of Life (QOL) than comparison groups, though some studies do report more optimistic findings. Higher autistic traits are also related to poorer QOL. However, the role of alexithymia in this relationship has not been explored.

**Method:**

A total of 163 participants (N = 53 autistic and N = 111 comparison) consented to take part; however, 30 participants were excluded due to missing data (who did not differ from those who were retained on age, gender, education, employment, or living status), leaving a final sample of 133 (N = 42 Autistic and 91 Comparison participants). Demographic information (including age, gender) was collected, alongside self-report measures of autistic traits, mental health, alexithymia, and QOL. We estimated regression models based on pre-registered analysis, and we conducted exploratory network analyses.

**Results:**

Alexithymic traits did not predict QOL when controlling for covariates. Depression significantly predicted Physical, Psychological, and Social QOL. When examining the impact of just alexithymic traits and autistic traits, both were significantly associated with Physical and Psychological QOL. For participants with a low depression score, the correlation between alexithymia and QOL was strong; suggesting that depression occludes the association between alexithymia and QOL. Network analyses suggested that depression and anxiety exert direct effects on Physical and Psychological QOL, whereas alexithymia scores may influence Physical QOL via autistic traits.

**Conclusion:**

In sum, depression is a pervasive negative predictor of multiple QOL domains. The role of alexithymia in predicting QOL dimensionally and categorically was not ruled out, given our exploratory analyses, we suggest that interventions which target alexithymia may positively impact QOL for those who score low on depressive symptoms.

## Highlights

• Associations between autistic and alexithymic traits, and quality of life have yet to be examined.
• We examined these associations in both autistic and comparison participants.
• Results suggest that alexithymia may have some indirect, negative impact on Quality of life.
• However, this is association is likely over-ridden by depression.

## 1 Introduction

Autism Spectrum Disorder (ASD, hereafter ‘autism’, ‘autistic people’, or ‘people on the autism spectrum’; Kenny et al., 2016) is a neurodevelopmental condition characterised by social communication difficulties and restricted interests and/or repetitive behaviours (American Psychological Association, 2013). One important research area for autistic people is Quality of Life (QOL). Self-reported QOL has been discussed by multiple authors as an important outcome for autistic adults (Burgess & Gutstein, 2007; Henninger & Taylor, 2013). Several recent empirical studies have supported this position. At the Internal Meeting for Autism, a Special Interest Group, consisting of researchers, autistic people, family members, suggested that ASD support in adulthood should investigate what promotes the QOL of autistic adults (Nicholas et al., 2017). A recent mental health priority setting study, co-produced with autistic people, collected data from an online survey of 136 autistic – and self-diagnosed – individuals. Results indicated that QOL was the most important outcome, receiving more endorsements than mental health, sleep, or interpersonal relationships, amongst others (Benevides et al., 2020). Thus, QOL is theoretically and empirically important to the lives of autistic people. This may be because it assesses the individual’s perception of how their life is going (Barcaccia et al., 2013), and is not necessarily strongly associated with traditional objective indicators (such as employment; Bishop-Fitzpatrick et al., 2016; Cummins, 2000; Mason et al., 2019), which many autistic people often struggle to achieve (Anderson et al., 2014; Henninger & Taylor, 2013; Mason et al., 2020; Ruble & Dalrymple, 1996).

QOL is conceptualised as a multi-faceted construct, assessed across numerous domains (Schalock, 2004; Whoqol Group, 1998). This study uses the World Health Organisation’s (WHO) conceptualisation of QOL as an “*individuals’ perceptions of their position in life in the context of the culture and value systems in which they live and in relation to their goals, expectations, standards and concerns* “(Whoqol Group, 1998, p551). We make use of the WHO Brief QOL measure (the WHOQOL-BREF; Skevington et al., 2004), which has been widely used with samples of autistic adults (Ayres et al., 2018). Moreover, this measure has recently been validated in a sample of autistic adults, which also led to the development of an autism-specific set of QOL items (McConachie et al., 2018). Moreover, a qualitative study comparing four nationalities (the United Kingdom, Australia, Singapore, and Argentina) revealed that the WHOQOL and autism-specific QOL items are viewed as important, although there may be within-country specific QOL issues (McConachie et al., 2020). A common finding is that autistic people often report poorer QOL when compared to comparison groups (e.g. Lawson et al., 2020; Lin & Huang, 2019; van Heijst & Geurts, 2015). However, it is important to note that some studies find autistic adults report QOL scores close to general population values (Hong et al., 2016; Moss et al., 2017), and that QOL reports at the individual level (rather than just comparing sample means) can be very good for autistic people (Oakley et al., 2021).

There is a growing consensus that autistic traits (as measured by, for example, the Autism Quotient; Allison et al., 2012) are continuously distributed in the general population (Constantino & Todd, 2003; Hoekstra et al., 2007), whereby diagnosed autistic individuals reside at the extreme tail of this distribution (Bralten et al., 2018). Autistic traits have also been used to investigate QOL in samples of non-autistic participants. The findings are broadly consistent with findings from samples of diagnosed autistic adults compared to non-autistic samples such that higher autistic traits have been associated with poorer self-reported QOL. Pisula et al. (2015) reported that – in a sample of 153 participants aged 19–38 – QOL was negatively correlated with autistic traits (with correlations ranging between -0.36 and -0.42). Likewise, Reed and colleagues identified that loneliness and social anxiety were mediators in this association, examining data from 413 university students aged 18–41; autistic traits were associated with greater loneliness and social anxiety, and both in turn were associated with poorer QOL (Reed et al., 2016).

Alexithymia is a construct both theoretically and empirically relevant to QOL; yet, alexithymia has not been explored to date in the autism QOL literature. Theoretically, it has been argued that affective response processes are an essential component of cognition (Smith et al., 2019). The WHOQOL-Bref contains several items which ask participants to rate how they feel about something (e.g. “To what extent do you feel your life to be meaningful?”, or “How safe do you feel in your daily life?”; World Health Organisation, 1998), therefore it is plausible that alexithymia is systematically associated with how these items are rated. This could plausibly be extended to other items on the measure, as they likely entail a cognitive appraisal and reliance on episodic memory. There is evidence of bias toward remembering fewer positive, and greater negative events for those high in alexithymic traits (Apgáua & Jaeger, 2019; Barchetta et al., 2021). Empirically, alexithymia has been found to be related to poorer QOL in a range of groups. A study of 5,418 general population participants found that alexithymia was significantly associated with poorer reported QOL on each of 15 domains of QOL even when controlling for depression (Mattila et al., 2009, 2010). In both Mattila and colleagues’ studies the alexithymia and QOL link was found even when controlling for age, gender, depression, anxiety, and somatic health. In clinical samples, a similar association between alexithymia and QOL has been observed. In a study of 60 Cirrhotic patients, for example, alexithymia predicted poorer mental QOL (as assessed by the SF-36) even when accounting for depression (Nardelli et al., 2013). Nekouei and colleagues found that alexithymia predicted poorer QOL in a sample of 398 patients with heart disease (their model also found that alexithymia predicted greater depressive symptoms; Nekouei et al., 2014). Alexithymia also predicted poorer mental health-related QOL in a sample of 205 females (both as a direct effect, and mediated by depressive symptoms) (Tesio et al., 2018). Whilst by no means exhaustive of this literature, these findings highlight that alexithymia may negatively impact on QOL in addition to the effect of mental and physical health problems.

Alexithymia is also more common in autism. For example, a recent meta-analysis of 15 studies (which compared diagnosed autistic people to comparison participants) reported that alexithymia was far more prevalent in the autism samples (c. 50 %), compared to the comparison samples (c. 5%) (Kinnaird et al., 2019; see also Albantakis et al., 2020 and Milosavljevic et al., 2016). So, it could be that higher autistic traits interact with alexithymia to have a more pronounced impact on QOL. Given the findings that autistic samples often report poorer QOL (with large effect sizes, compared to normative data; Mason et al., 2018) and alexithymia is more common in autistic samples, alexithymia may act as a second-hit on QOL for autistic people. We hypothesise this double-hit may also be the case in those undiagnosed but with high autistic traits.

Given the foregoing, it seems plausible to hypothesise that both autistic traits and alexithymic traits will be associated – negatively – with QOL. However, there are a range of factors to consider. First, there are reported gender differences in alexithymia. A meta-analysis of 41 studies found a small effect (Hedge’s g = 0.22), with males reporting greater alexithymia scores (Levant et al., 2009). Second, depression is associated with alexithymia in both clinical and community samples (Foran & O’Leary, 2013; Honkalampi et al., 2000). Likewise, anxiety is associated with alexithymia. For example, in a study of 226 individuals admitted to an emergency department those with an anxiety disorder scored higher for alexithymia than those without such a disorder, even after controlling for age, gender, education, and level of physical illness (Marchesi et al., 2000). Anxiety and depressive symptoms are correlated with autistic traits in the general population (Liew et al., 2015; Rosbrook & Whittingham, 2010), and anxiety and depressive disorders commonly co-occur with autism (Hollocks et al., 2019). Therefore, it is essential to account for the inter-relationships between gender and anxiety and depressive symptoms when considering a link between alexithymia, autistic traits, and QOL.

This study sought to address the putative connection between alexithymia, autistic traits and QOL using pre-registered regression analyses. In addition, network analysis is emerging as a powerful way to examine variables that may be associated in non-linear ways (e.g. bi-directional associations; Borsboom and Cramer, 2013). In network analysis, variables are represented as nodes (which can be item-level, or a total score) and the relationships between nodes are called edges (Hevey, 2018). Edges convey information about the strength of an association between two nodes; an edge can be positive or negative (positive correlation and negative correlation, respectively) and weighted or unweighted (a weighted edge has a thickness proportional to the strength of the association; an unweighted edge shows only the presence of an association; Hevey, 2018). This approach has been used in studies of QOL with samples of autistic adults. Deserno et al. (2017) found that subjective well-being, as assessed by the question “How happy are you?” was positively associated with societal contribution and satisfaction with social contacts, and negatively associated with the number of physical health problems. Further work, asking clinicians to judge causal associations between self-report network variables found a good degree of concordance; suggesting purported factors underlying QOL are amenable to self-report and proxy-observation (Deserno et al., 2020). Yet, to date, network analysis has not been used in relation to autistic traits in a mixed sample of those with and without an autism diagnosis and QOL variables. Thus, we undertook exploratory network analyses of the variables used in the present study, with particular focus on the relationship between alexithymia, autistic traits, mental health and QOL.

In summary, this study sought to address the following questions, using linear regression analyses: how are autistic traits and alexithymic traits associated with QOL (when controlling for appropriate covariates)? Is the association between autistic traits and QOL mediated by alexithymia? And when considered categorically (i.e. diagnosed autistic people versus general population participants), is there a difference in QOL domains? Finally, we undertook exploratory network analyses to explore how age, gender, alexithymia, autistic traits, QOL, and mental health are interconnected.

## 2 Method

### 2.1 Participants

Participants were eligible for inclusion if they met the following criteria: 1) age 18 years or older; and 2) resident of the United Kingdom. A total of 163 eligible participants completed the survey. The mean age of the sample was 31.9 (SD = 13.1, range = 18–72), with 41 males, 109 females, and 13 who reported another gender identity. The difference between the AQ-totals for those who self-identified as autistic and those with a formal diagnosis was non-significant (Welch’s t-test, t(39.3) = 1.23, p = .226). Therefore, the 30 participants who reported having a formal ASD diagnosis and 22 who self-identified as autistic are hereafter referred to as the ASD group. The remaining 111 participants were designated as the comparison group.

The sample comprised diverse levels of education; 1.5 % of the sample reported having no formal qualifications, 29.3 % reported having secondary/high-school qualifications, 33.1 % reported having a bachelor’s degree, and 36.1 % reported having a postgraduate degree. The majority – 59.4 % – of the sample were currently employed, and 27.1 % were currently unemployed, the remainder were unable to work due to ill health (9.8 %) or were retired (3.8 %). There were a lot of different responses to the living status question. Broadly, 66.3 % lived independently (i.e. alone, with a partner or spouse, with a roommate or friend, or with children). The remainder lived with parents, siblings, or in supported living/a retirement home. For a detailed breakdown see Supplementary Material 1.

For 30 participants the total proportion of missing data was greater than 20 % (ranging from 23 % to 94 %). These participants were excluded from the present analyses, leaving a final sample of 133 (81.6 % of the total sample). The proportion of participants with complete data above 80 % did not vary by group (i.e. ASD vs Comparison; χ^2^(1) = 0.04, p = .852). Those with 80 % or more data were compared to those with less than 80 % complete data on demographic variables. There were no significant differences on any key variables (age, gender, education, employment, or living arrangements), therefore, this smaller sample of 133 participants was considered representative of the full sample (see Supplementary Material 2a for analyses).

### 2.2 Procedure

Participants were recruited via social media platforms (e.g. Twitter, Reddit). The study advert informed participants about the aim of the study, and provided a link to complete the study on Qualtrics (www.qualtrics.com). All participants completed an online consent form before being taken to the study questions. Please note, data were collected between March and June of 2020. This time period coincided with the early COVID-19 lockdowns in the United Kingdom (UK), and we included only adults (aged 18 years or older) who were resident in the UK, to avoid any confound of country/lockdown status during the time of data collection.

### 2.3 Measures

Participants completed a range of demographic information at the beginning of the survey. These data were not used in the present analysis (apart from age and gender) and are presented in Supplementary Material 1 to characterise the sample.

Participants also completed a range of self-report measures of psychological constructs, of which the following were used in the present analyses:

Autism Quotient-10 (AQ-10; Allison et al., 2012). The AQ-10 is a 10-item measure designed to screen for autistic traits in adults. Each item is scored on a 4-point Likert scale. Items measure behaviours or cognitions characteristic of ASD such as attention to detail (“I usually concentrate more on the whole picture, rather than the small details”) and social difficulties (“I find it difficult to work out people’s intentions”). Greater scores indicate more autistic traits, and a score of 6 or more is taken to indicate a potential need for a full ASD assessment. The AQ-10 was originally derived from the full Autism Quotient (AQ-50) (Allison et al., 2012). The measurement properties of the AQ-50 and AQ-28 (a shortened AQ measure) in general population samples has recently been reported to be inconsistent (and it has been recommended that subscale scores are used, rather than the total score; English et al., 2020). Similarly, the AQ-10 initially had promising psychometrics (for example, high internal consistency and a good ability to discriminate autistic from non-autistic participants; Allison et al., 2012). However, the measurement properties of the AQ-10 have recently been questioned (Bertrams & Shah, 2021; Taylor et al., 2020), although a recent largescale population sample study suggested adequate validity for measuring individual differences in autistic traits (Lundin et al., 2019).

Toronto Alexithymia Scale (TAS-20; Bagby et al., 1994). This is a 20-item self-report measure of alexithymia. Each item is measured on a 5-point Likert scale. The measure comprises three subscales; difficulty identifying feelings (DIF; “I am often confused about what emotion I am feeling”), difficulty describing feelings (DDF; “It is difficult for me to find the right words for my feelings”), and externally orientated thinking (EOT; “I prefer to analyse problems rather than just describe them”). Higher scores indicate more alexithymic traits. A cut-off of 61 or more indicates categorical assignment of alexithymia. A recent study, published after pre-registering these analyses, reported that the TAS-20 did not evidence good psychometric properties in a large sample of autistic adults (743 cognitively able adults, Williams & Gotham, 2021). Williams and Gotham (2021) propose a reduced 8-item measure of alexthymia, derived from the TAS-20. We provide analyses with both the TAS-20 and the 8-item version.

World Health Organisation’s Quality of Life Questionnaire-Brief^1^ (WHOQOL-BREF; Skevington et al., 2004). This is a 26-item measure of QOL comprised of two global questions (about satisfaction with overall QOL and with physical health) and 4 domains of QOL. Physical QOL (e.g. “How much do you need any medical treatment to function in your daily life?”), Psychological QOL (e.g. “How much do you enjoy life?”), Social QOL (e.g. “How satisfied are you with your personal relationships?”), and Environment QOL (e.g. “How satisfied are you with your access to health services?”). Each item is scored on a 5-point Likert scale. A higher score indicates better self-reported QOL. This measure has recently been validated for use with autistic adults (McConachie et al., 2018).

Patient Health Questionnaire-9 (PHQ-9; Arroll et al., 2010). This is a 9-item measure derived from the full PHQ. Each item has a 4-point Likert response, with each item scored 0 (not at all) to 3 (nearly every day). Each item asks the participant to rate the extent of their problems over the past two weeks (e.g. “Trouble falling or staying asleep, or sleeping too much”). Greater scores indicate greater levels of depression. A recent confirmatory factor analysis reported this measure was suitable for use with autistic adults (Arnold et al., 2020).

Generalised Anxiety Disorder-7 (GAD-7; Löwe et al., 2008). This is a 7-item measure designed to capture the main diagnostic features of GAD. Each item is scored on a 4-point Likert scale from 0 (not at all) to 3 (nearly every day). Each item asks the participant to rate the extent of their problems over the past two weeks (e.g. “Being so restless it is hard to sit still”). Greater scores indicate greater levels of anxiety. To our knowledge, this measure has not been validated for use with autistic people.

### 2.4 Ethics

This study was approved by the Research Ethics Committee at King’s College London (reference HR-19/20-17129).

### 2.5 Analysis plan

This analysis was registered on the Open Science Framework website (https://osf.io/), after collecting data via the online survey platform Qualtrics. Briefly, the first analysis was to regress each QOL domain onto the data in three blocks in a hierarchical manner. Block one consisted of demographic data and mental health scores (PHQ and GAD total scores). Block two added alexithymia traits and autistic traits. Finally, block three consisted of the interaction between alexithymia traits and autistic traits. The second, categorical, analysis compared the ASD vs Comparison groups and high/low alexithymia groups (controlling for age, GAD-7 and PHQ-9). The alpha level was set to 0.05, and p-values were adjusted using the Holm-Bonferroni method (Abdi, 2010; Boustani, 2020). Details of planned and exploratory analyses can be found at https://osf.io/79kxr/. After completing the analysis, we noted that the correlations between depression and anxiety were particularly high (we picked these constructs a-priori, as they are associated with both alexithymia and autism). To ensure the regression models were not affected by multicollinearity, we examined the variance inflation factor (VIF) and tolerance statistic for each variable in each model (age, gender, and total scores on: alexithymia, autistic traits, depression, anxiety). The VIF was below 5 for each variable in each model, and the tolerance statistic was above 0.2 (depression and anxiety scored highest for VIF and tolerance); indicating minimal concerns about multicollinearity (Field, 2013).

Exploratory analyses were conducted after the planned analyses. These were: categorical analyses comparing those who scored high, or low, on alexithymia and QOL controlling for covariates, categorical analyses were re-run without covariates comparing correlation coefficients between alexithymia and QOL for those with low PHQ scores and the remainder of the sample. The regression analyses were re-run using an 8-item alexithymia measure derived from the TAS-20 (Williams & Gotham, 2020). For reasons of space, these analyses are included in Supplementary Material 6.

A network analysis approach was used to explore how the study variables were related; variables are entered as nodes (which can represent any entity or variable) and nodes can be connected by edges (which represent an association between nodes; Dalege et al., 2017). Interpreting a network is aided by centrality indices, which give an indication of the importance of any given node to its network (Hevey 2018). Nodes are placed according to the Fruchterman-Reingold algorithm, such that strongly connected nodes are placed closer together (Kossakowski et al., 2016). The strength of a node indicates the direct influence a node has on the network (as it is the sum of the absolute edge values connected to a given node; Dalege et al., 2017). Closeness conveys how likely it is that information “travels” through a network; as edges can be construed as paths connecting nodes, stronger nodes indicate an easier path from one node to another (Dalege et al., 2017; Kossakowski et al., 2016). Finally, betweenness indicates the number of shortest paths a given node lies on (Dalege et al., 2017).

As gender was included in the network as a dichotomous variable, we first ran a Mixed Graphical Model to estimate the network (which is suitable for mixed data; Hevey 2018). As gender (and age) were disconnected from the network (i.e. gender and age had no edges) we re-ran the network analysis after removing gender (as this was the only dichotomous variable). For this second network, we used the Gaussian Graphical Model, with the EBICglasso procedure. Applying the EBICglasso accounts for the fact that correlation networks will often be fully connected, and this procedure reduces small, weak edge estimates to zero (Hevey 2018), leading to a sparser network.

To assess the stability of the edges in the network, we used non-parametric bootstrapping to generate 95 % confidence intervals for each edge estimate (Epskamp et al., 2018). To estimate the stability of the centrality indices we used the case-dropping subset bootstrap approach (Epskamp et al., 2018). This procedure randomly drops a proportion of participants from the data set, re-estimates the centrality indices, and then estimates the correlation between the order of the centrality indices in the original network and case-dropped network (the proportion of removed participants is increased up to 75 %). A sharp decrease in this correlation indicates unstable estimates (Epskamp et al., 2018).

## 3 Results

### 3.1 Dimensional approach: factors associated with QOL

Table 1A shows the descriptive statistics for each IV and DV for the total sample, and by group (see Table 1B for descriptive statics by gender and diagnostic group, along with effect size comparisons). We include Cronbach’s alpha for the full sample. For reasons of space, we exclude the Cronbach’s alpha for each subgroup. Briefly, all alpha’s were above .600 for the formal diagnosis group (except for the WHOQOL-BREF Social domain, alpha = .572); the same was true for the comparison group (except for the TAS-20 EOT domain, alpha = .519); for the self-identified group, all subscales and total scales had a Cronbach’s alpha greater than .600. Correlations between each continuous variable can be found in Table 2.

**Table 1A:** Descriptive statistics for each study measure.

	Full sample (N = 133)				ASD sample (N = 42)						Comparison sample (N = 91)		
					Formal diagnosis (N = 24)			Self-identified (N = 18)					
Measure	Mean	SD	Range	Cronbach’s alpha	Mean	SD	Range	Mean	SD	Range	Mean	SD	Range
AQ-10													
Total score	4.55	3.00	0−10	.875	7.79	2.25	3−10	6.94	2.19	3−10	3.23	2.30	0−10
N (%) meeting cutoff	45 (33.8)				19 (79.2)			13 (72.2)			13 (14.3)		

TAS-20													
Total score	52.43	13.24	24−85	.895	61.65	11.11	42−85	56.69	9.64	38−78	49.15	13.07	24−74
DIF^a^	18.32	6.97	7−35	.897	23.21	5.56	13−35	21.02	5.24	12−31	16.50	6.86	7−33
DDF^b^	15.08	5.10	5−25	.866	18.29	4.39	9−25	16.28	3.23	12−24	14.00	5.19	5−25
EOT^c^	19.03	4.24	10−30	.532	20.14	4.98	12−30	19.39	4.20	11−27	18.66	4.02	10−30

PHQ-9													
Total score	11.35	6.86	0−27	.898	13.56	6.71	2−25	14.09	5.90	3−23	10.22	6.84	0−27

GAD-7													
Total score	9.98	6.02	0−21	.916	12.17	5.67	2−20	12.28	5.50	5−21	8.98	5.99	0−21

WHOQOL-BREF domains^d^													
Physical	64.77	18.93	4−100	.800	51.79	19.07	14−75	54.37	19.54	4−86	70.25	16.23	18−100
Psychological	47.06	21.66	0−88	.868	38.89	19.72	4−71	42.36	19.56	4−71	50.14	22.01	0−87.5
Social	50.06	23.87	0−100	.740	43.40	23.95	0−100	46.30	28.18	0−83	52.56	22.76	0−91.7
Environmental	63.98	20.03	9−100	.849	56.90	20.85	19−91	55.38	26.73	9−97	67.55	17.37	16−100

a Difficulty identifying feelings.

b Difficulty describing feelings.

c Externally orientated thinking.

d Indicates transformed score (scaled from 0 to 100).

**Table 1B:** Descriptive statistics by group (ASD or Comparison) and gender (male, female, other gender identity), with effect size comparisons.

	ASD sample			Comparison sample			Effect size comparisons (Hedge’s g)				
Measure	Male (N = 12) [1]	Female (N = 21) [2]	Other (N = 9) [3]	Male (N = 18) [1a]	Female (N = 69) [2a]	Other (N = 4) [3a]	[1] vs [3]	[2] vs [3]	[1a] vs [3a]	[2a] vs [3a]	[3] vs [3a]
	Mean (SD)	Mean (SD)	Mean (SD)	Mean (SD)	Mean (SD)	Mean (SD)					
AQ-10											
Total score	7.67 (2.10)	6.62 (2.09)	9.00 (2.00)	3.47 (1.84)	3.07 (2.35)	4.75 (3.20)	−0.62	−1.12	−0.59	−0.69	−1.65

TAS-20											
Total score	60.72 (9.18)	59.02 (11.73)	59.11 (11.01)	53.39 (13.27)	47.65 (12.93)	60.50 (6.76)	0.15	−0.01	−0.62	−1.00	0.13
DIF^a^	22.01 (4.28)	22.54 (5.91)	22.00 (6.38)	16.89 (7.23)	16.22 (6.90)	19.50 (4.65)	0.00	0.09	−0.36	−0.48	−0.39
DDF^b^	17.92 (4.50)	16.62 (4.03)	18.67 (3.24)	15.11 (4.17)	13.31 (5.26)	20.75 (2.22)	−0.18	−0.52	−1.38	−1.42	0.65
EOT^c^	20.79 (5.05)	19.86 (4.71)	18.44 (3.94)	20.39 (4.58)	18.11 (3.60)	20.25 (6.65)	0.49	0.31	0.03	−0.56	0.35

PHQ-9											
Total score	11.55 (6.44)	14.86 (5.33)	14.26 (8.07)	10.83 (7.29)	10.05 (6.84)	10.50 (6.13)	−0.36	0.09	0.04	−0.07	−0.46

GAD-7											
Total score	10.58 (5.81)	12.38 (5.17)	14.25 (5.97)	8.67 (5.48)	8.94 (6.19)	11.00 (5.89)	−0.60	−0.34	−0.41	−0.33	−0.50

QOL domains^d^											
Physical	64.88 (13.69)	47.96 (19.45)	48.41 (18.99)	72.22 (10.72)	70.24 (17.66)	61.61 (7.36)	0.98	−0.02	0.99	0.49	0.73
Psychological	48.61 (17.44)	36.90 (19.91)	37.50 (19.87)	51.62 (20.82)	50.42 (22.47)	38.54 (20.80)	0.58	−0.03	0.60	0.53	0.05
Social	50.00 (26.35)	48.02 (22.81)	29.63 (27.67)	49.07 (26.02)	53.99 (21.75)	43.75 (27.53)	0.73	0.74	0.20	0.46	0.48
Environmental	71.35 (18.93)	54.91 (20.62)	39.24 (23.29)	67.71 (20.51)	67.44 (17.09)	68.75 (6.25)	1.48	0.71	−0.05	−0.08	1.36

**Table 2:** Pairwise correlations for self-report measures.

		1	2	3	4	5	6	7	8	9
1	Physical^a^	1.000								
2	Psychological^a^	.650***	1.000							
3	Social^a^	.419***	.595***	1.000						
4	Environment^a^	.571***	.545***	.498***	1.000					
5	AQ-10	−.489***	−.348***	−.201*	−.159	1.000				
6	TAS-20	−.348***	−.415***	−.279*	−.156	.632***	1.000			
7	PHQ-9	−.635***	−.770***	−.499***	−.512***	.360**	.454***	1.000		
8	GAD-7	−.500***	−.717***	−.397***	−.505***	.369***	.445***	.829***	1.000	
9	Age	.014	.125	−.116	−.066	.034	−.084	−.198*	−.119	1.000

a WHOQOL-BREF domains.

To simplify the regression analyses, which included gender, those who reported a gender identity other than male or female were excluded – see Supplementary Material 2b for statistical comparison between the males and females used in the regression and the full sample of 133 participants. Robust Minimum Covariance Determinant (MCD) analysis identified multivariate outliers for each domain of the WHOQOL-BREF (Leys et al., 2018; Verardi and Dehon, 2010). MCD is derived from Mahalanobis distances and is used to detect multivariate outliers (Leys et al., 2018). However, Mahalanobis distance can be overly sensitive and so a conservative selection criterion is recommended (Tabachnik & Fidell, 2013). Thus, we opted to only exclude a data point as an outlier if the MCD distance was significant at the <.05 level (Tabachnik & Fidell, 2013). This led to the following number of participants being identified as outliers: Physical QoL (N = 0, 0.0 %), Psychological QoL (N = 1, 0.8 %), Social QoL (N = 1, 0.8 %), and Environment QoL (N = 0, 0.0 %). These participants were excluded from each regression model. Outliers for the set of all study variables were identified for the purposes of calculating correlations (N = 0 outliers, 0.0 %).

Demographic and mental health variables (model 1) explained variance in the Physical, Psychological, and Social QOL domains. Specifically, higher depression scores were associated with poorer QOL in all three domains. Higher anxiety scores were associated with poorer Psychological QOL, and increasing age was associated with poorer Social QOL. When adding alexithymic traits and autistic traits (model 2), depression remained a significant predictor of Physical, Psychological, Social and Environment QOL. Both higher depression scores and autistic traits were associated with poorer Physical QOL. However, model 2 did not explain significantly more variance than model 1 for Psychological or Social QOL. Adding the interaction term (model 3) did not explain more variance than the preceding model for any domain. Yet, greater depression scores were associated with poorer Physical, Psychological, Social and Environment QOL; autistic traits were associated with poorer Physical QOL. The interaction term between autistic traits and alexithymic traits was significant for the Psychological domain. The results of the regression analyses can be found in Table 3A (regression weights, p-values, 95 % confidence intervals and Table 3B (variance explained). When we repeated our analyses using only the 8 TAS items suggested by Williams and Gotham (2021) (argued to be a more valid alexithymia measure), the results were unchanged (and can be found in Supplementary Material 6).

**Table 3A:** Regression coefficients, p-values, and 95 % confidence intervals for each regression model of the WHOQOL-BREF domain.

	Physical			Psychological			Social			Environment		
	B	p-value	CI	B	p-value	CI	B	p-value	CI	B	p-value	CI
Model 1												
Age	−0.18	.225	[-0.37,0.02]	−0.05	.617	[-0.23,0.14]	**−0.39**	**.015**	[-0.66,-0.12]	−0.24	.128	[-0.46,-0.02]
Gender	−4.66	.256	[-10.67,1.35]	−3.11	.566	[-8.82,2.60]	2.14	1.000	[-6.06,10.35]	−4.92	.435	[-11.57,1.72]
PHQ	**−2.07**	**<.001**	[-2.77,-1.38]	**−1.78**	**<.001**	[-2.44,-1.11]	**−2.03**	**<.001**	[-2.99,-1.08]	−1.00	.435	[-1.77,-0.23]
GAD	0.35	.375	[-0.43,1.14]	**−0.99**	**.033**	[-1.74,-0.23]	0.26	1.000	[-0.82,1.34]	−0.72	.435	[-1.58,0.15]

Model 2												
Age	−0.13	.339	[-0.31,0.05]	−0.05	1.000	[-0.23,0.14]	***−0.40***	***.020***	[-0.67,-0.13]	−0.24	.180	[-0.45,0.02]
Gender	−7.11	.056	[-12.79,-1.45]	−4.41	.560	[-10.28,1.47]	1.56	1.000	[-6.95,10.07]	−4.16	.696	[-11.02,2.70]
PHQ	**−1.94**	**<.001**	[-2.59,-1.29]	***−1.66***	***<.001***	[-2.34,-0.99]	***−1.96***	***<.001***	[-2.94,-0.98]	**−1.07**	**.048**	[-1.86,-0.29]
GAD	0.56	.339	[-0.17,1.28]	−0.90	.105	[-1.65,-0.14]	0.30	1.000	[-0.79,1.40]	−0.78	.324	[-1.66,0.10]
AQ	**−2.65**	**<.001**	[-3.77,-1.52]	−0.45	1.000	[-1.61,0.71]	0.43	1.000	[-1.25,2.11]	−0.04	.954	[-1.40,1.32]
TAS	0.15	.339	[-0.10, 0.39]	−0.12	1.000	[-0.38,0.14]	−0.19	1.000	[-0.56,0.18]	0.14	.696	[-0.15,0.44]

Model 3												
Age	−0.10	.270	[-0.28,0.08]	−0.01	1.000	[-0.20,0.18]	−0.33	.075	[-0.60,-0.06]	−0.20	.335	[-0.42,0.01]
Gender	−7.95	.030	[-13.56,-2.35]	−5.00	.368	[-10.82,0.83]	0.44	1.000	[-7.88,8.77]	−5.06	.532	[-11.88,1.77]
PHQ	**−1.99**	**<.001**	[-2.63,-1.36]	***−1.75***	***<.001***	[-2.42,-1.08]	***−2.12***	***<.001***	[-3.08,-1.16]	***−1.13***	***.035***	[-1.90,-0.35]
GAD	0.66	.216	[-0.06,0.44]	−0.76	.264	[-1.52,-0.00]	0.56	1.000	[-0.53,1.64]	−0.67	.532	[-1.55,0.21]
AQ	**−3.14**	**<.001**	[-4.32,-1.96]	−0.91	.438	[-2.13,0.32]	−0.43	1.000	[-2.19,1.32]	−0.56	.532	[-2.00,0.87]
TAS	0.19	.242	[-0.05,0.44]	−0.06	1.000	[-0.33,0.19]	−0.09	1.000	[-0.46,0.28]	−0.19	.532	[-0.11,0.49]
AQ*TAS^a^	0.08	.088	[0.01,0.15]	0.08	.264	[0.00,0.16]	***0.15***	***.048***	[0.04,0.26]	0.09	.264	[0.00,0.18]

a Denotes interaction term between AQ-10 total score and TAS-20 total score. Significant results are highlighted in bold (note, all p-values have been adjusted using the Holm-Bonferroni correction). Regression coefficients in italics refer to significant effects in models that do not explain a significant amount of variance more than the preceding model.

**Table 3B:** Significance tests for regression analyses, along with variance explained.

	R^2^_adj_^a^	p-value	ΔR^2^_adj_ (p-value)
Physical QOL			
Model 1	41.1%	<.001	–
Model 2	50.6%	<.001	9.5% (<.001)
Model 3	52.4%	<.001	1.8% (.022)

Psychological QOL			
Model 1	61.1%	<.001	–
Model 2	61.4%	<.001	0.3% (.225)
Model 3	62.5%	<.001	1.1% (.098)

Social QOL			
Model 1	27.9%	<.001	–
Model 2	27.3%	<.001	−0.6% (.596)
Model 3	31.1%	<.001	3.8% (.017)

Environment QOL			
Model 1	29.7%	<.001	–
Model 2	29.2%	<.001	−0.5% (.546)
Model 3	31.2%	<.001	2.0% (.087)

a Denotes adjusted R^2^; b R^2^ values are reported as, due to poor fit, adjusted R^2^ was negative.

Planned regression analyses of just autistic traits and alexithymic traits (with their interaction) were carried out. For Physical QOL those with higher autistic traits reported poorer QOL (B=-2.99). Higher alexithymic traits were associated with poorer Psychological and Social QOL (B=-0.56 and B=-0.46 respectively). However, the inclusion of the interaction of autistic traits and alexithymic traits did not significantly improve the explanation of variance in QOL for the Physical or Psychological domains (see Supplementary Materials 3a and 3b for the regression results and variance explained).

### 3.2 Categorical analyses: high and low autistic traits, high and low alexithymic traits

A 2 (group; ASD vs. Comparison) x 2 (alexithymia; high vs. low) x 4 (WHOQOL-BREF domains) MANCOVA (with age, depression, and anxiety scores as covariates) was estimated. The Omnibus test was significant (Wilk’s Δ = .228, F(24.0, 462.8) = 9.38), p < .001). There was a significant main effect of being in the ASD group on QOL (Wilk’s Δ = .879, F(4.0, 122.0) = 4.19), p < .001). Depression total score and anxiety total score were significant covariates (p < .001). However, there was no main effect of having high alexithymia traits (Wilk’s Δ = .936, F(4.0, 122.0) = 2.09), p = .228), nor was there a significant interaction between diagnosis group and alexithymic trait groups (Wilk’s Δ = .948, F(4.0, 122.0) = 1.69), p = .228). Age was a non-significant covariate. Follow-up analyses showed that the autistic group reported significantly lower Physical QOL (57.4, SD = 2.31) compared to the Comparison group (68.4, SD = 1.45; p < .001), controlling for age, depression, and anxiety. However, there were no other significant QOL differences between the ASD and Comparison group (all p > .05). Note, this pattern of results was the same when comparing these analyses were conducted with high (versus low) autistic traits (instead of diagnostic group). We re-ran this analysis without covariates and the results were the same. We observed a main effect of diagnosis on Physical QOL (such that the autistic participants reported poorer Physical QOL), but there was no effect of alexithymia group, nor was there a significant interaction.

A final exploratory one-way MANOVA with gender (3 levels; male, female, other gender identity) and QOL (Physical, Psychological, Social, Environment) was estimated. The model was significant (Wilk’s Δ = .885, F(8, 254.0) = 2.00, p = .048). However, post-hoc analyses revealed no significant QOL differences between genders.

### 3.3 Exploratory analyses: correlations between QOL and TAS-20, based on depression scores

Given the pervasive effect of depression on QOL, we decided to examine the correlations between TAS-20 scores and each QOL domain after separating participants based on PHQ-9 score. In brief, we opted to use a PHQ-9 score of 5 or less to identify those with low depressive symptomatology (called ‘very low PHQ’) the remaining participants were categorised as the ‘remaining PHQ’ group. In the autism group, there were 5 who were classified as ‘very low PHQ’ and 37 who were in the ‘remaining PHQ’ group; in the comparison sample, 24 participants were classified as ‘very low PHQ’ and 67 were classified into the ‘remaining PHQ’ group. Briefly, we found that alexithymia score and QOL were significantly, and highly, correlated in the very low PHQ group for Physical, Psychological, and Social QOL (rs -.734, -.436, and -.569 respectively). These correlation coefficients were significantly different (i.e. lower) compared to the remaining PHQ group for the Physical and Social domains. This pattern was less clear when we repeated this analysis by group (ASD and Comparison), where the correlations were mostly non-significant (likely due to the small N in these groups). The only significant correlation coefficient differences was between the PHQ groups for the Physical QOL domain (r_ASD_=-.658, r_COMP_=-.261, z = 2.08) The results are detailed in Supplementary Material 5.

### 3.4 Exploratory network analyses

**Fig. 1:**
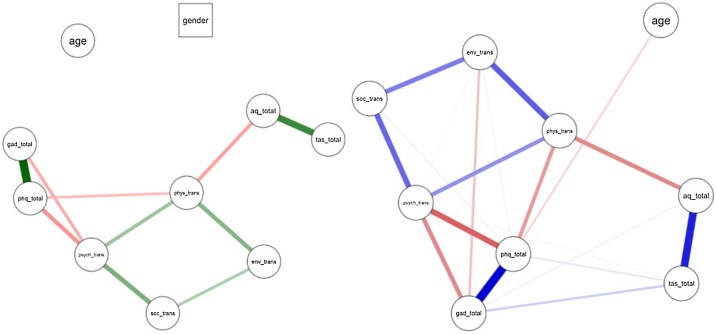
Network analyses. Left panel shows the Mixed Graphical Method (MGM), and the right panel shows the Gaussian Model. Variables are represented by nodes, and lines connecting nodes – edges – indicate associations between nodes. Blue/green indicates a positive association, and red indicates a negative association. Abbreviations: age, gender, “env”- environment QOL, “soc”- social QOL, “psy”- psychological QOL, “aq”- AQ-10 total, “phy”- physical QOL, “tas”- TAS-8 total, “dep”- PHQ-9 total score, “anx”- GAD-7 tota. (For interpretation of the references to colour in this figure legend, the reader is referred to the web version of this article).

The left panel of Fig. 1 shows the Mixed Graphical Model (MGM) for the study variables. In this graph, each node represents a study variable. Green paths indicate positive associations and red paths indicate negative associations - the thickness of the path indicates the strength of the association (thicker paths indicate stronger connections; Kossakowski et al., 2016). As can be seen from Fig. 1, left panel, all 4 QOL domains were placed together, and had positive interrelations. Depression and anxiety were both strongly related, and in turn were negatively associated with Psychological QOL; depression was negatively associated with Physical QOL. Autistic traits were positively associated with alexithymia, and negatively associated with Physical QOL. Of note, alexithymia was independent of depression and anxiety in this network. Finally, of note, age and gender were not connected to this network. This suggests that associations between these two variables and the remaining nodes are accounted for by other variables in the network.

As a result, we estimated a network excluding gender (Fig. 1, right panel; note, for this network we included those with any gender identity). This network was broadly similar to the MGM graph; all QOL domains were positively interrelated, as were depression and anxiety, and autistic traits and alexithymia remained strongly associated with each other and some QOL domains. Both depression and anxiety were negatively associated with Psychological QOL, and depression was negatively associated with Physical QOL. In this second analysis, alexithymia was positively – albeit weakly – associated with both depression and anxiety. Also of note is the negative association – again, albeit weak – between alexithymia and Psychological QOL. Finally, autistic traits were strongly negatively associated with Physical QOL, and weakly positively associated with anxiety. In this network, age was negatively associated with depression. For centrality and stability measures, see Supplementary Material 4.

## 4 Discussion

This study sought to explore the links between the constructs of alexithymia, autistic traits, and QOL. Based on studies in the general population and other clinical groups showing that alexithymia predicts poorer QOL, we predicted that alexithymia would be associated with poorer QOL, and that autistic traits would interact with alexithymic traits. These hypotheses were not supported. Based on a-priori regression analyses, depression was a strong negative predictor of Physical, Psychological, and Social QOL; autistic traits only significantly predicted poorer QOL in the Physical QOL domain. Alexithymic traits were not significantly associated with any QOL domain in the models that also included depression and anxiety. Only depression scores predicted Environment QOL. However, exploratory network analyses revealed that alexithymia showed weak (positive) associations with depression and anxiety, and a weak negative association with Psychological QOL. Alexithymia and autistic traits were very strongly associated in the network, suggesting a close association between these constructs. When we examined group differences (diagnosed/self-identified autistic and comparison, and alexithymic versus non-alexithymic) we only found a main effect of diagnosis group. Specifically, autistic participants reported lower Physical QOL (controlling for age, depression, and anxiety).

These findings are somewhat consistent with previous, trait-based studies. Specifically, depression was also the strongest predictor of QOL for autistic adults (using the same WHOQOL measure) in a recent EU-Aims study (Oakley et al., 2021). Oakley and colleagues examined QOL in a sample of 192 adults (N = 106 on the autism spectrum) aged 18–30 years and found, for the autism group, that depression significantly predicted Physical, Psychological, and Social QOL domains of the WHOQOL-BREF. Neither autistic traits nor anxiety predicted QOL. Although this study, and others, have found group based QOL differences (with autistic people reporting poorer QOL; Jennes-Coussens et al., 2006; Kamio et al., 2013; Kamp-Becker et al., 2010; Lin, 2014; Lin & Huang, 2019; Oakley et al., 2021; van Heijst & Geurts, 2015), there is evidence that *within* group QOL variability is not always explained by degree of autistic traits (for a meta-analysis reaching this conclusion, see Kim & Bottema-Beutel, 2019). However, both Mason et al. (2018) and Lawson et al. (2020) found autistic traits and mental health predict poorer QOL for autistic people. One explanation for this discrepancy could be methodological; given the wealth of measures of QOL used in autism research (Roestorf et al., 2019), and the different measures of mental health, direct comparisons of between-study effects are very difficult. Alternatively, each study used different predictors in their respective regression models. Thus, estimates within each regression model may be biased if an unmeasured predictor is related to QOL (and correlates with measured predictors; Kline, 2015). While some studies assess the same domain as a predictor of QOL (e.g. education), the assessment of that domain is typically not measured on the same scale (cf. Lawson et al., 2020 and Mason et al., 2018). Moreover, even studies with similar sets of variables, do not use identical sets of predictors in their models.

Another consideration is the way the current regression models were specified. As mental health problems are often associated with poor QOL (Khanna et al., 2014; Knüppel et al., 2018; Mason et al. 2018, 2019) we entered depression and anxiety as covariates to try to isolate the effects of alexithymia on QOL. However, the association between mental health and alexithymia is itself complex. First, as reported by Morie et al. (2019) in a sample of 64 autistic adults, alexithymia and emotional regulation serially mediated the association between autistic traits and both depression and anxiety. Further, Albantakis et al. (2020) found that both autistic traits and alexithymic traits predicted depression in a sample of autistic adults and a comparison sample. However, the direction of the association differed between groups; for the ASD group TAS-20 predicted depression when controlling for autistic traits, but the converse was not true, while in the comparison group, both autistic traits and alexithymia traits predicted depression irrespective of the order of entry in the regression model. This is consistent with the findings of Bloch et al. (2021), who found that alexithymic traits were a significant predictor of depression symptoms, more so than autistic traits.

Motivated by this complexity, we augmented the present regression analyses with an exploratory network analysis. The network identified that the QOL domains clustered, as might be expected from the construct (and as reported at the item and domain level with the SF-36; Kossakowski et al., 2016). Moreover, connections between depression and both Physical QOL and Psychological QOL were identified, and alexithymia was related to Psychological QOL. Intriguingly, and contrary to previous studies that identified a strong association between alexithymia and depression (Honkalampi et al., 2000; Lipsanen et al., 2004), the nodes for alexithymia and depression showed only a weak association. However, the centrality measures of betweenness and strength – the degree to which a node lies on the shortest path between any two nodes, and the number of connections incident on a node respectively (Hevey, 2018; see Supplementary Material 4) – were stronger for depression than for alexithymia. Thus, whilst alexithymia and depression may exert somewhat independent effects, the effect of depression on QOL may be somewhat larger than that of alexithymia.

When considered categorically, we only identified a main effect of autism on QOL. Specifically, autism was associated with poorer Physical QOL compared to the comparison group (no other domain differences or interactions were significant). The main effect of being in the autism group for only one domain is inconsistent with most previous studies, which tend to report that diagnosed autistic samples report significantly lower QOL than comparison samples across most domains (Ayres et al., 2018, van Heijst & Geurts 2015, Lawson et al., 2020, Lin et al., 2019). One explanation for the lack of associations reported here, is a genuine lack of association between autism, alexithymic traits, and QOL. This seems plausible, given the regression coefficients for alexithymia were close to zero in the models. A second plausible explanation for the lack of associations could be that the effects of individual differences in trait alexithymia are occluded by the presence of differences in state depression/depressive symptoms. Alexithymia is considered to be a trait-like construct which is largely stable (e.g., de Haan et al., 2014; Luminet et al., 2001), albeit that some state like variability has been reported (e.g. de Haan et al., 2014; Šago & Babić, 2019). Hence, as will be discussed below, fluctuating depressive symptoms may be more salient and the effect of alexithymia therefore attenuated. A third plausible explanation for the lack of associations reported here is the context in which this study occurred. Our survey was carried out during COVID-19, specifically during and after the first lockdown in the United Kingdom. The mean QOL scores reported here (with a sample comprised of 70 % typically developing participants) are notably lower than previously reported normative data (Skevington & McCrate, 2012) for Physical, Psychological, and Social QOL (Hedge’s *g* = 0.38, 1.10, and 0.86 respectively), but not Environment QOL (Hedge’s *g* = 0.05; see Supplementary Material 7). Indeed, the negative effect of COVID-19 on QOL has been reported internationally (e.g. in Italy, China, Saudi Arabia, and Vietnam; Melo-Oliveira et al., 2021). One possible mechanism for this is that, under lockdown, people’s roles change (e.g. working from home, and having to manage domestic life in parallel); multitasking is cognitively demanding and can be stressful, which may impact QOL (Ferreira et al., 2021). Perhaps acting in tandem with this are individual differences in a range of psychological resources. For example, the psychological demands of lockdown may tax faculties such as resilience and ability to cope with adversity (Shek, 2021). A range of mechanisms likely explain the effect of COVID-19 on QOL, operating to different degrees at different developmental stages; for example, younger people may be more impacted by restrictions on social contact (Ravens-Sieberer et al., 2020). Given the wealth of evidence that, overall, autistic people often report poorer QOL, it seems more likely that statistical power, combined with the impact of COVID-19, are the main explanations for the present null findings. Future studies, with larger samples, would be well placed to further explore this relationship, as suggested below.

Overall, it is tempting to conclude that depression is more strongly related to QOL than either autistic traits or alexithymic traits, yet the specific relationship between depression, alexithymic traits, and QOL is likely to be nuanced. Despite alexithymia being strongly associated with depression (Li et al., 2015), it is still possible to score highly for alexithymia and low for depression. For such individuals, it is perhaps premature to conclude that alexithymia is not a relevant predictor of QOL. To examine this possibility, we compared those who scored very low on depression, with those with higher scores. We found that alexithymia was strongly negatively correlated with QOL in those very low in depression (we leave elaboration of these findings for Supplementary Material 5, as the sample sizes are small and any conclusions necessarily tentative), but future research should investigate how alexithymia and QOL are linked for those with very low/no depressive symptomology.

Our main focus in this paper was not gender. Gender was included in the models to control for reported gender differences in the constructs of interest, as noted in the Introduction. However, we did report gender differences for males, females, and those reporting a different gender identity for the autism and the comparison group (we thank an anonymous reviewer for suggesting this). Briefly, those who reported a gender identity other than male or female tended to report more autistic traits, more mental health problems, and lower QOL for many, but not all of the scales (see Table 1B). However, we must emphasise that we opted not to compare these data inferentially, in part because of the very small number of participants in these gender by diagnostic groups. Thus, this comparison of effect sizes may be informative for future hypothesis testing but must be interpreted with caution.

A final point to consider is how the present results from adults with high autistic traits apply to those with an autism diagnosis. Given autistic people typically score highly for autistic traits, the present findings may contribute to understanding the poorer QOL of autistic people. However, it must also be borne in mind that high autistic traits may not necessarily equal autism (in the same way that autistic symptoms do not always indicate autism; Lord & Bishop, 2021). Thus, these findings do need replication in a large sample of diagnosed autistic people. If these findings prove applicable to autistic people, we can identify the following factors related to the QOL of autistic people. First, as commonly reported (Khanna et al., 2014; Mason et al., 2018, 2019; McCauley et al., 2020) QOL is negatively impacted by depression, even when accounting for emotional awareness (as operationalised here by alexithymia). Second, we can see that alexithymia may exert negative effects on QOL for autistic people. This suggests that QOL may be improved for autistic people by targeting alexithymia (in those who score highly on the TAS-20). Third, based on the network analysis, the mechanism by which alexithymia impacts QOL may be different from the mechanism of the impact of depression and anxiety on QOL. In this analysis, depression directly affected Physical QOL, and both depression and anxiety exerted effects directly on Psychological QOL (and anxiety directly affected Environment QOL). However, alexithymia did not directly affect any QOL domain, but was positively associated with autistic traits (which in turn were negatively associated with Physical QOL). Thus, depression and anxiety may be proximal causes of poorer Physical and Psychological QOL, whereas alexithymia may act more distally via autistic traits (and to some extent depression and anxiety, given the weak positive associations between alexithymia and depression and anxiety). Indeed, network psychopathology is predicted on the notion that symptoms interact in feedback loops, beyond simple A-causes-B relationships. Our findings suggest, tentatively, that alexthymia traits may have a particular role in QoL when depression states are absent.

### 4.1 Strengths/limitations

A significant strength of this study is that two of the instruments, the WHOQOL-BREF and PHQ-9, have been validated for use with autistic participants (Arnold et al., 2020 and McConachie et al., 2018 respectively). Thus, the reported QOL scores are likely to accurately reflect each participant’s perceived QOL. This is also true of the depression scores; thus, the associations between QOL and depression are unlikely to be due to measurement issues and likely reflect the real association between depression on QOL. As noted in the results section, the psychometric properties of the TAS-20 for autistic adults have recently been questioned (Williams & Gotham, 2021). When we repeated our analyses with the suggested briefer TAS-8, the findings were in line with those of the TAS-20 data, but more associations were identified (e.g., a positive association between female gender and QOL). This is an intriguing finding, given that autistic females often report poorer QOL (Bishop‐Fitzpatrick et al., 2017; Kamio et al., 2013), yet not always in the social domain (Mason et al., 2018). The use of the TAS-20 may have obscured the effect of sex in the regression analyses of this study. So, we would suggest future studies attempt to replicate the present findings using the perhaps more valid 8-item TAS measure. A second strength of this study is the broad range of demographics within the sample. The participants span a broad range of ages, educational levels, and employment statuses. Third, there were no differences between those who completed more than 80 % of the survey and those who did not on those demographics. Nor were there any differences (apart from a greater proportion of other gender identities in the ASD group) between those in the regression models, and those not included. This suggests that the sample used in the analyses is likely representative of the total sample, and the results from this sample are unlikely to be biased by participant characteristics unique to those who took part. However, in light of a recent paper by Rubenstein and Furnier (2021) we cannot be sure how far these findings generalise to the broader autism population; our findings would certainly, at most, be constrained to those capable of completing self-report measures online. Indeed, our sample was more educated and living more independently than samples of autistic people reported elsewhere (Mason et al., 2020; Steinhausen et al., 2016).

Whilst not a controllable limitation, the recent COVID-19 outbreak adds complexity when interpreting the results of the present study, as outlined above. Future studies examining QOL before, during and after the COVID-19 pandemic could help explore QOL changes that may have occurred due to COVID. A second limitation is that this study has a small sample size, and the study may not be sufficiently powered to detect the effect of alexithymia on QOL when also accounting for depression. However, our finding that depression was a negative predictor of multiple QOL domains, with autistic traits explaining no further variance, is broadly in line with the larger study by Oakely et al. (2020).

## 5 Conclusion

This study found that depression was most strongly related to QOL in this sample; autistic traits were predictive of Physical QOL, but alexithymic traits did not significantly predict QOL. Yet, several findings did emerge that nuance this conclusion and may advance the literature. First, planned regression analyses using only autistic and alexithymic traits did indeed find that alexithymia and autistic traits predicted some QOL domains (Physical and Psychological). Exploratory network analyses revealed that QOL domains were positively inter-related as expected; importantly, alexithymia was weakly related to Psychological QOL, whereas depression was more associated with Physical and Psychological QOL. In exploratory analyses, we found that QOL was strongly correlated with alexithymic traits for those with low depressive symptoms (however, given the very small samples sizes we only suggest this an avenue for future research). Thus, whilst targeting depression may improve QOL for many, there may well be a vulnerable sub-group with poorer QOL who are not depressed but are alexithymic. Future research should attempt to replicate and extend this novel finding.

## CRediT authorship contribution statement

Both **David Mason** and **Francesca Happé** conceptualised the study and both designed the data collection methodology. **David Mason** drafted the initial data analysis plan, which was reviewed and approved by **Francesca Happé**. **David Mason** managed the data collection, data curation, and conducted all analyses; both **David Mason** and **Francesca Happé** interpreted the findings. **David Mason** drafted the manuscript, which was reviewed, finalised, and approved by both authors.

## Declaration of Competing Interest

The authors report no declarations of interest.

## Footnotes

1 Note, we did administer the Autism-Specific QOL items alongside the measure (McConachie et al., 2018). However, we did not also administer the WHOQOL Disabilities module. As such, we have not reported the ASQOL data because the full set of required measures was not used (Rodgers, 2021).
